# Does Cell Size Impact Chloroplast Genome Size?

**DOI:** 10.3389/fpls.2017.02116

**Published:** 2017-12-14

**Authors:** David R. Smith

**Affiliations:** Department of Biology, University of Western Ontario, London, ON, Canada

**Keywords:** *Acetabularia*, cell size, DNA content, genome size, plastid genome

## Abstract

There is a strong positive relationship between nuclear genome size and cell size across the eukaryotic domain, but the cause and effect of this relationship is unclear. A positive coupling of cell size and DNA content has also been recorded for various bacteria, suggesting that, with some exceptions, this association might be universal throughout the tree of life. However, the link between cell size and genome size has not yet been thoroughly explored with respect to chloroplasts, or organelles as a whole, largely because of a lack data on cell morphology and organelle DNA content. Here, I speculate about a potential positive scaling of cell size and chloroplast genome size within different plastid-bearing protists, including ulvophyte, prasinophyte, and trebouxiophyte green algae. I provide examples in which large and small chloroplast DNAs occur alongside large and small cell sizes, respectively, as well as examples where this trend does not hold. Ultimately, I argue that a relationship between cellular architecture and organelle genome architecture is worth exploring, and encourage researchers to keep an open mind on this front.

Today, I am still intrigued by the massive range in chloroplast and mitochondrial DNA (ptDNA and mtDNA) size as I was as an undergraduate student. Indeed, organelle genome length can differ by more than three orders of magnitude (from a few kilobases to many megabases) across the eukaryotic domain (Smith and Keeling, 2015). And, like with other kinds of genome, this difference in size is largely due to the presence or absence of non-coding DNA. Over the years, I—and many others—have investigated the puzzle of organelle genome size variation from different angles, exploring, for example, the roles of mutation rate, genetic drift, and natural selection on ptDNA and mtDNA expansion (Smith, 2016). But this work has provided no clear answers, except for the realization that the forces influencing organelle genome architecture are complex, multifaceted, and can vary within and among lineages.

Recently, I’ve been asking myself the question: is there a relationship between cell size and chloroplast genome size? This might sound like both a sensible and a silly question. Sensible because there is an extensive body of literature showing a strong positive association between cell size and nuclear genome size in diverse eukaryotes, from plants to animals to protists (Gregory, 2001a; Beaulieu et al., 2008; Connolly et al., 2008); and the same trend holds for various bacteria (Cavalier-Smith, 1982; Shuter et al., 1983; Sabath et al., 2013). However, some might also consider the question silly for at first glance there appears to be no obvious connection between the diameter of a cell and the length of a chloroplast genome. Case in point: 95% of the more than 1,500 completely sequenced ptDNAs from land plants fall within the narrow size range of 120–170 kb despite the fact these genomes come from a remarkable diversity of species and lineages, including ones with drastically different cellular architectures.

But things get a bit more interesting when looking at algae. For instance, the unicellular prasinophyte green alga *Ostreococcus tauri* is the smallest free-living eukaryote ever observed (∼0.8 μm in diameter) (Courties et al., 1994) and, sure enough, it has one of the smallest known ptDNAs from a photosynthetic organism (71.7 kb, >80% coding, and one intron) (Robbens et al., 2007). Likewise, its close relative *Micromonas commoda* is also incredibly tiny (<2 μm in diameter) and has a highly reduced ptDNA (72.6 kb) (Worden et al., 2009). In fact, picoeukaryotes as a whole appear to have a propensity for miniaturized chloroplast genomes (Lemieux et al., 2014), as well as for very small mitochondrial and nuclear genomes (Derelle et al., 2006; Robbens et al., 2007; Worden et al., 2009).

At the other end of the spectrum sits the gargantuan green alga *Acetabularia acetabulum* (mermaid’s wineglass). This single-celled marine ulvophyte is so massive it can be seen with the naked eye (**Figure 1**), making it among the largest of all unicellular eukaryotes (1–10 cm) (Mandoli, 1998). It also boasts one the biggest chloroplast genomes on record (∼2 Mb) (Palmer, 1985), but unfortunately the huge number of repeats in this ptDNA have hindered sequencing efforts (de Vries et al., 2013), and its exact size remains unknown (the same is also true for the mitochondrial and nuclear genomes). In addition to a massive cell and chloroplast genome, *A. acetabulum* also has a huge nucleus (Mandoli, 1998), but the size and number of its chloroplasts are unremarkable (Shephard, 1965). Ulvophytes from the order Cladophorales, such as *Boodlea composita*, *Dictyosphaeria cavernosa*, and *Valonia ventricosa*, can also have large cells (easily visible by the naked eye) (**Figure 1**), and have recently been shown to have highly fragmented, single-stranded linear ptDNAs, which are partly characterized and potentially very big (Del Cortona et al., 2017).

**FIGURE 1:**
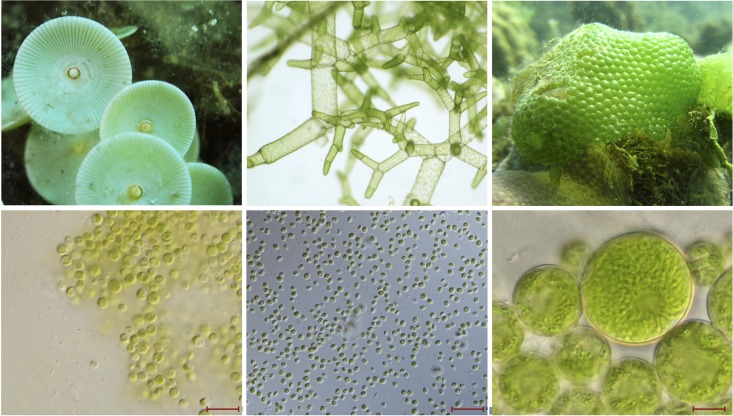
Images of macro- and micro-green algae. **(Top)** Left to right, large ulvophyte green algae, which are visible by the naked eye: *Acetabularia* sp. (image by Albert Kok), *Boodlea composita* (image by Frederik Leliaert), and *Dictyosphaeria cavernosa* (image by Frederik Leliaert). **(Bottom)** Left to right, images from Veselá et al. (2011): picoprasinophyte *Picosystis salinarum* (scale bar 10 μm), picoplanktonic trebouxiophyte *Choricystis* sp. (scale bar 20 μm), and trebouxiophyte *Dictyochloropsis splendida* (scale bar 10 μm).

For the longest time, *A. acetabulum* was the only act in town with an enormous chloroplast genome, but explorations of poorly studied red algal groups have uncovered other species and lineages with prodigious ptDNAs. One of these species is the unicellular rhodellophycean *Corynoplastis japonica*, whose plastid genome weighs in at a whopping 1.13 Mb, is >80% non-coding, and has 311 introns (Muñoz-Gómez et al., 2017), making it the biggest, most intron-rich ptDNA yet sequenced. The cell size of *C. japonica*, although not as extraordinary as *A. acetabulum*, is still quite large (18–33 μm in diameter) (Yokoyama et al., 2009), and an order of magnitude larger than those of *O. tauri* and *M. commoda*. The rhodophyte *Bulboplastis apyrenoidosa* is a close relative of *C. japonica* and it, too, has an immense plastid genome (0.61 Mb, 220 introns) (Muñoz-Gómez et al., 2017) as well as a moderately large cell (Kushibiki et al., 2012). Red algae can also have small, compact ptDNAs. The ultra-tiny unicell *Cyanidioschyzon merolae* (2 μm in diameter) has perhaps the most compact plastid genome of all photosynthetic eukaryotes (∼95% coding) (Ohta et al., 2003), as well as very coding dense nuclear and mitochondrial genomes (Ohta et al., 1998; Matsuzaki et al., 2004).

Based on this anecdotal evidence, one could be forgiven for thinking that ptDNA size is positively associated with cell size, at least in certain algae. The problem is that this is not an easy hypothesis to test. Plastid genome size data are lacking for many major algal groups, especially those with “complex” plastids (Burki, 2017), and in some cases when ptDNA size data are available, detailed cell diameter statistics are missing.

One algal lineage for which we are gaining more and more plastid genome data each year and for which there are significant information on cell size are prasinophyte green algae—again, the class to which *O. tauri* and *M. commoda* belong. Complete ptDNAs sequences are now available for at least 14 different prasinophytes, spanning six of the major clades (Lemieux et al., 2014; Turmel and Lemieux, 2018). Most of these species are picoplanktonic—organisms with a diameter of less than 3 μm (**Figure 1**)—and, not surprisingly, their ptDNAs are extraordinarily small and coding-dense, averaging about 80 kb in length. The smallest plastid genome from this cohort belongs to Prasinophyceae sp. CCMP 1205 (64.3 kb) (Lemieux et al., 2014), and although this species has not been formally described, it appears to have a very small cell (Le Gall et al., 2007). Conversely, non-picoplanktonic prasinophytes have much larger genomes and cell sizes (Lemieux et al., 2014). The freshwater prasinophyte *Nephroselmis olivacea*, for example, has a 200.8 kb plastid genome (Turmel et al., 1999) and a cell size that greatly exceeds that of its picoprasinophyte close relatives: 8–10 μm in diameter, and sometimes much larger (Suda et al., 1989).

Similar trends emerge from trebouxiophyte green algae. Plastome size in the Trebouxiophyceae has generally been unimpressive, but researchers have started identifying species with unexpectedly large (and small) ptDNAs (Turmel et al., 2015). In some instances, big ptDNAs are associated with big cells, and vice versa. As noted by others, the ptDNAs of picoplanktonic and nanoplanktonic taxa (**Figure 1**), such as *Choricystis minor* (94.2 kb), *Marsupiomonas* sp. NIES 1824 (94.3 kb), *Pedimonas minor* (98.3 kb), and *Marvania geminata* (108.5 kb), are the smallest among explored trebouxiophytes (Turmel et al., 2015). Species with larger cells (**Figure 1**), however, can have much longer ptDNAs. Take *Dictyochloropsis reticulata* (also called *Symbiochloris handae*), which can have cells as large as 26 μm in diameter (Škaloud et al., 2016) and houses a 289.4 kb plastid genome (Turmel et al., 2015). Similarly, *Pleurastrosarcina brevispinosa* (also called *Chlorosarcina brevispinosa*) is approximately 25 μm in diameter when mature (Chantanachat and Bold, 1962) and has a ptDNA in excess of 295 kb, the second largest currently found in the Trebouxiophyceae (Turmel et al., 2015). The largest plastome in the class belongs to *Prasiolopsis* sp. SAG 84.81 (306 kb), but cell morphology data are unavailable for this strain.

Of course, one can find examples where these trends do not hold. The phagomixotrophic prasinophyte *Cymbomonas tetramitiformis* is far from small (∼10 um in diameter) (Maruyama and Kim, 2013) but has a minute ptDNA (∼85 kb) (Satjarak et al., 2016). Most diatom and dinoflagellate algae do not have particularly large ptDNAs but can have very big cells (Finkel et al., 2009). The colonial chlamydomonadalean alga *Tetrabaena socialis* has a large ptDNA (>405 kb) (Featherston et al., 2016), but its cell size is unexceptional (∼10 μm in diameter). Its close multicellular relative *Volvox carteri* has an even longer plastome (∼525 kb) (Smith and Lee, 2010) and, likewise, the average somatic cell size is only 5–9 μm, but the asexual reproductive cells (gonidia) are much larger (13–90 μm) (Kirk et al., 1993). Closely affiliated with the Chlamydomonadales is another order—the Chaetopeltidales—with huge ptDNAs. The chaetopeltidalean species *Koshicola spirodelophila* and *Floydiella terrestris* have giant plastomes (384.9 and ∼520 kb, respectively), but unlike their chlamydomonadalean counterparts they do have hefty cells (up to 32 μm long and 55 μm wide) (Brouard et al., 2010; Watanabe et al., 2016).

Then there is the non-photosynthetic green algal genus *Polytomella* whose members appear to have completely forfeited their plastid genomes (Smith and Lee, 2014) but do not have overly small cells (about 10–15 μm in diameter) (Pringsheim, 1955). However, the forces responsible for extreme plastid genome reduction and outright plastome loss are arguably different than those involved in the expansion and contraction of non-coding ptDNA (Figueroa-Martinez et al., 2017). For all we know, the ancestral ptDNA of *Polytomella* species might have had an expanded architecture before being jettisoned. The colorless green alga *Polytoma uvella*, which is closely related to *Polytomella* (the two lineages lost photosynthesis independently of one another), has the most expanded ptDNA ever found in a non-photosynthetic species (∼230 kb, 75% non-coding DNA) (Figueroa-Martinez et al., 2017). *P. uvella* is also relatively large for a plastid-bearing colourless protist: up to 18 μm long and 14.5 μm wide (Moewus and Moewus, 1959).

So, after considering the points described above, I’m still left scratching my head, wondering if there isn’t, for some species, a link between cell size and chloroplast genome size. For now, detailed data on cell morphology and ptDNA length are too sparse to rigorously test such a hypothesis, nor would I necessarily want to argue in favor of one just yet. My aim is to simply point out that the relationship between cellular architecture and organelle genome architecture is worth exploring, and to encourage researchers to keep an open mind on this front.

Some readers might have noted that I skimmed over an important point regarding previous work on cell size and genome size: it is not so much that big cells have big genomes (and vice versa) but that big cell have big DNA contents (Gregory, 2001b). Because nuclear genomes often have low ploidy levels (e.g., haploid or diploid), the DNA content of nuclei is strongly positively correlated with genome size, and thus both these parameters scale positively with cell size (Gregory, 2001b). However, in highly polyploid systems it is possible to have a high DNA content occurring alongside a small or moderately sized genome. The gram-positive bacterium *Epulopiscium fishelsoni* exemplifies this point. It has a 3.8 Mb circular genome, which is present in about 200,000 copies, resulting in a DNA content in excess of 750 Gb (Mendell et al., 2008). *E. fishelsoni* is also one of the largest known prokaryotes, growing up to 600 μm in length (Bresler et al., 1998).

Chloroplasts and mitochondria are polyploid. The number of genomes per organelle can vary throughout a lifecycle, across tissues in multicellular organisms, and from species to species, but it is usually quite high (>10), sometimes extremely so (Kuroiwa et al., 1981; Raven, 2015; Cole, 2016). The cryptophyte alga *Guillardia theta*, for instance, carries between 130 and 260 copies of its 121.5 kb plastid genome (Hirakawa and Ishida, 2014), and some land plants have nearly a 1000 copies of the ptDNA in their chloroplasts (Raven, 2015, and reference therein). Even more impressive are the mitochondria from diplonemid and kinetoplastid protists, which can contain 1000s of copies of a fragmented mitochondrial genome leading to hyper-inflated mtDNA contents, whereby the mitochondria can contain more DNA than the nucleus (David et al., 2015). The mitochondrion of the kinetoplastid *Perkinsella* strain Gill-NOR1/I is so inflated with DNA that it takes up nearly the entire cell and can be >10 μm in diameter (David et al., 2015). Similarly, there are species with giant chloroplasts an elevated ptDNA contents (e.g., *Vicia faba*), and plastid genome copy number has long been known to rise in parallel with increases in chloroplast volume, and to go down alongside a reduction in volume (Kuroiwa et al., 1981). It is noteworthy in this context that the ptDNA copy number in the chloroplasts of *Acetabularia* is estimated to be small (1–4) (Kuroiwa et al., 1981).

Organelle ploidy level is not an easy parameter to calculate (Rooney et al., 2015), and can be influenced by many different factors, including evolutionary forces acting on genes involved in organelle biogenesis and organelle–nuclear gene interactions (Cole, 2016). Consequently, we still have a lot to learn about the DNA content and genome length of chloroplasts and mitochondria and how they might be connected to other cellular features, including cell size and organelle volume. It has been shown that the number of mitochondria and chloroplasts per cell can influence the rate of intracellular DNA transfer from organelles to the nucleus (Smith et al., 2011) and from plastids to mitochondria (Smith, 2011). Environmental conditions are also thought to influence organelle DNA architecture. For example, plastid genomic compaction in the endolithic ulvophyte seaweed *Ostreobium quekettii* and the palmophylalean green alga *Verdigellas peltata* is thought to have been shaped primarily by adaptation to low light conditions (Marcelino et al., 2016).

Much has been written about the processes responsible for the well-established link between DNA content and cell size and whether it is adaptive or non-adaptive (Cavalier-Smith, 1982; Gregory, 2001b; Lynch, 2007). Some have argued that genomic streamlining and its strong association with a small cell size and a high growth rate provides a metabolic advantage in certain situations (Hessen et al., 2010), and it has been noted that streamlined ptDNAs in picoplanktonic and nanoplanktonic chlorophytes could confer a selective advantage (Turmel and Lemieux, 2018). Others have suggested that DNA may have quantitative non-coding functions, potentially acting as a “skeleton” within the cell (Cavalier-Smith, 1982). And there is always the strong possibility that the forces influencing genome size are purely non-adaptive (Lynch, 2007). Finally, the likelihood that cell size is directly connected to important traits, such as photosynthetic rate, are also important considerations when evaluating potential relationships between cell size and genome size. If there does turn out to be a relationship between cell size and organelle DNA length/content in certain systems, it will likely only add further mystery and complexity to the long-standing debate about the evolution of genome size.

## Author Contributions

The author confirms being the sole contributor of this work and approved it for publication.

## Conflict of Interest Statement

The author declares that the research was conducted in the absence of any commercial or financial relationships that could be construed as a potential conflict of interest.
